# A multicellular organism with embedded cell clusters from the Ediacaran Weng'an biota (Doushantuo Formation, South China)

**DOI:** 10.1111/ede.12210

**Published:** 2016-11-10

**Authors:** John A. Cunningham, Kelly Vargas, Federica Marone, Stefan Bengtson, Philip C. J. Donoghue

**Affiliations:** ^1^ Department of Palaeobiology and Nordic Center for Earth Evolution Swedish Museum of Natural History Stockholm 10405 Sweden; ^2^ School of Earth Sciences University of Bristol Life Sciences Building 24 Tyndall Avenue Bristol BS8 1TQ England; ^3^ Swiss Light Source Paul Scherrer Institute Villigen 5232 Switzerland

## Abstract

Three‐dimensional analyses of the early Ediacaran microfossils from the Weng'an biota (Doushantuo Formation) have focused predominantly on multicellular forms that have been interpreted as embryos, and yet they have defied phylogenetic interpretation principally because of absence of evidence from other stages in their life cycle. It is therefore unfortunate that the affinities of the various other Doushantuo microfossils have been neglected. A new conical fossil that is preserved at a cellular level is described here. The fossil contains distinct cell clusters that are characterized and analysed in three dimensions. These clusters are often exposed at the specimen surface, and the fossil preserves many hemispherical craters that are interpreted as positions where clusters have left the organism. The cell clusters may be either reproductive propagules or infesting organisms. Similar clusters are found in a variety of Doushantuo organisms including putative animal embryos and algae.

## INTRODUCTION

Microfossils from the ca. 570 Ma Weng'an Doushantuo biota provide a rare snapshot of soft‐bodied, unicellular and multicellular life in the prelude to the rich Phanerozoic fossil record. A diverse array of purported animals has also been reported from this assemblage in South China, though these interpretations have almost invariably proven contentious. For example, *Tianzhushania* (which we consider a senior synonym of *Megasphaera*, *Yinitianzhushania*, *Parapandorina*, and *Megaclonophycus*, see below) has been interpreted as an embryo of a variety of animals (Xiao et al. (1998); Chen et al. (2014), though see Huldtgren et al. (2011)), but also as a giant sulphur bacterium (Bailey et al. (2007), though see Xiao et al., 2007 and Cunningham et al. (2012)), a cyst‐forming protist (Huldtgren et al. (2011, 2012), though see Xiao et al. (2012)), or an alga (Butterfield 2011; Chen et al. 2014; Zhang and Pratt 2014). The principal cause of this equivocation is the lack of confident identification of later stages in the life cycles of these organisms. Competing interpretations exist because our knowledge of the diversity of organisms represented in the biota and their life cycles remains incomplete. The predominant focus of three‐dimensional analyses of Doushantuo fossils has been on *Tianzhushania*; the identity of later developmental stages of these organisms is unclear. Although later embryonic and post‐embryonic stages have been proposed (Xiao et al. 2007; Huldtgren et al. 2011; Chen et al. 2014), they have not been widely accepted and, thus, little is established concerning the life histories of these and other Doushantuo organisms. Here we describe a conical Doushantuo organism based on data from Synchrotron Radiation X‐ray Tomographic Microscopy (SRXTM) and Environmental Scanning Electron Microscopy (ESEM). This fossil contains near‐spherical cell clusters that may have been reproductive propagules or exogenous organisms with a parasitic, mutualistic or commensal relationship with the conical organism. We assess the implications for understanding the lifecycles and affinities of the Doushantuo organisms.

## MATERIALS AND METHODS

The specimen was recovered from rocks collected from the Upper Phosphorites of the Datang Quarry, Weng'an, Guizhou Province, China. It was extracted by dissolution of the host carbonate in 6–10% acetic acid and manual picking under a binocular microscope. The SRXTM study (Donoghue et al. 2006) was carried out at the X02DA (TOMCAT) beamline (Stampanoni et al. 2006) of the Swiss Light Source at the Paul Scherrer Institute, Villigen, Switzerland. The specimen was analysed using a beam energy of 17.5 keV and a 20× objective, which resulted in voxel dimensions of 0.325 μm. Images were recorded at 1501 stepwise increments through a rotation of 180°. These projection images were processed and rearranged into dark‐ and flatfield‐corrected sinograms and were then reconstructed using a gridding procedure and a highly optimized routine based on the Fourier transform method (Marone and Stampanoni 2012). In the resulting tomograms brighter regions correspond to regions of higher X‐ray attenuation and reflect denser regions of the sample. The tomographic datasets supporting this article are available at http://dx.doi.org/10.5523/bris.1gew13jeleafa1bwcp0x0klu2h. The tomographic data were analysed and measured using Avizo software. The ESEM analyses were carried out on a Philips XL‐30 microscope operating in backscatter mode at approximately 14 kV. The specimen is housed at the Swedish Museum of Natural History, specimen number X 5331, as are two further fossils containing cell clusters (X 4447 and X 5357) that are illustrated for comparison.

Volumetric measurements of cell clusters within the conical fossil and their component cells were made in Avizo. Two‐dimensional measurements of cell clusters were also compared for published and unpublished specimens (Fig. 5). These measurements came from three sources: (i) published measurements from Chen et al. (2014); (ii) measurements from clusters made from published images in Xiao et al. (1998), Xiao et al. (2004), Xiao et al. (2014), and Chen et al. (2014); and (iii) measurements from tomograms of the conical specimen presented here, of other previously unpublished specimens and of a cluster in a tubular fossil figured in Cunningham et al. (2015). The measurements were taken from two‐dimensional slices in order to be comparable to the published data. The 3‐D measurements were rounded to the nearest cubic micron (though the precision is likely to be lower in reality) and the 2‐D measurements to the nearest micron. Where mean measurements are used, the mean was rounded to one decimal place. All measurements are available in Table S1.

The taxonomy of the embryo‐like fossils from the Weng'an Biota is not widely agreed upon. Here we consider *Megasphaera*, *Yinitianzhushania*, *Parapandorina*, and *Megaclonophycus*, as junior synonyms of *Tianzhushania*, that merely reflect developmental and/or preservational differences of the same organism (Yin et al. (2004), Huldtgren et al. (2011), but see Xiao et al. (2014) for an alternative taxonomic interpretation). Chen et al. (2014) described “Megaclonophycus”‐stage specimens containing cell clusters (their “matryoshkas”), which they also interpret as a later developmental stage of the embryo‐like fossils.

## DESCRIPTION

**Figure 1:**
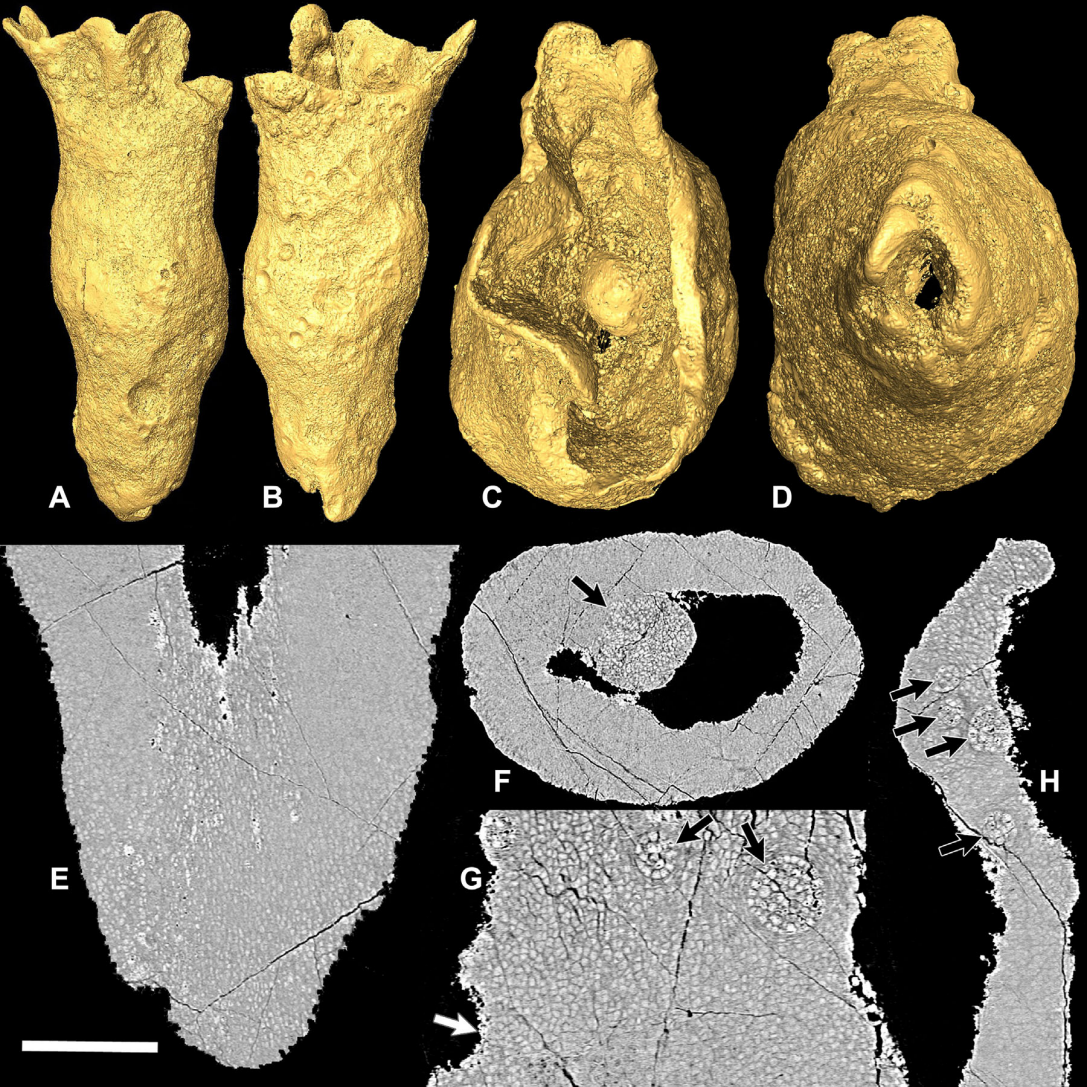
Reconstructions and SRXTM slice data of the new conical organism (X 5331). (A,B) Surface renderings in lateral view. (C) Surface rendering showing the aperture. (D) Surface rendering showing the apex. (E) Longitudinal slice showing cells with long‐axes aligned near the apex. (F) Transverse slice showing a large cell cluster (black arrow). (G,H) Longitudinal slices showing cell clusters (black arrows) and hollows (white arrow). Scale bar: (A, B) 300 μm, (C,D) 120 μm, (E) 70 μm, (F) 110 μm, (G) 35 μm, and (H) 75 μm.

**Figure 2:**
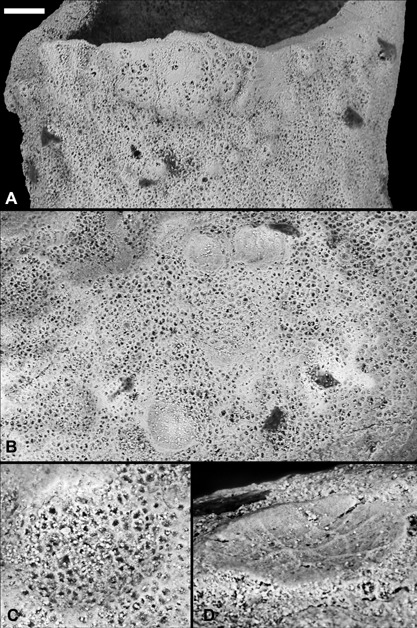
ESEM images of the surface of the new conical organism (X 5331). The polygonal pits that make up the entire surface are cells in which the outer surface is not present. There are numerous depressions and bulges on the surface. (A) The region around the aperture showing bulges. (B) View of a region of the surface of the specimen showing hemi‐spherical craters. (C) Detail of a crater with cells visible on the concave surface. (D) Detail showing an oblique view of a relatively smooth crater; note the polygonal network of ridges. Scale bar: (A) 40 μm, (B) 35 μm, (C) 16.5 μm, and (D) 12.5 μm.

**Figure 3:**
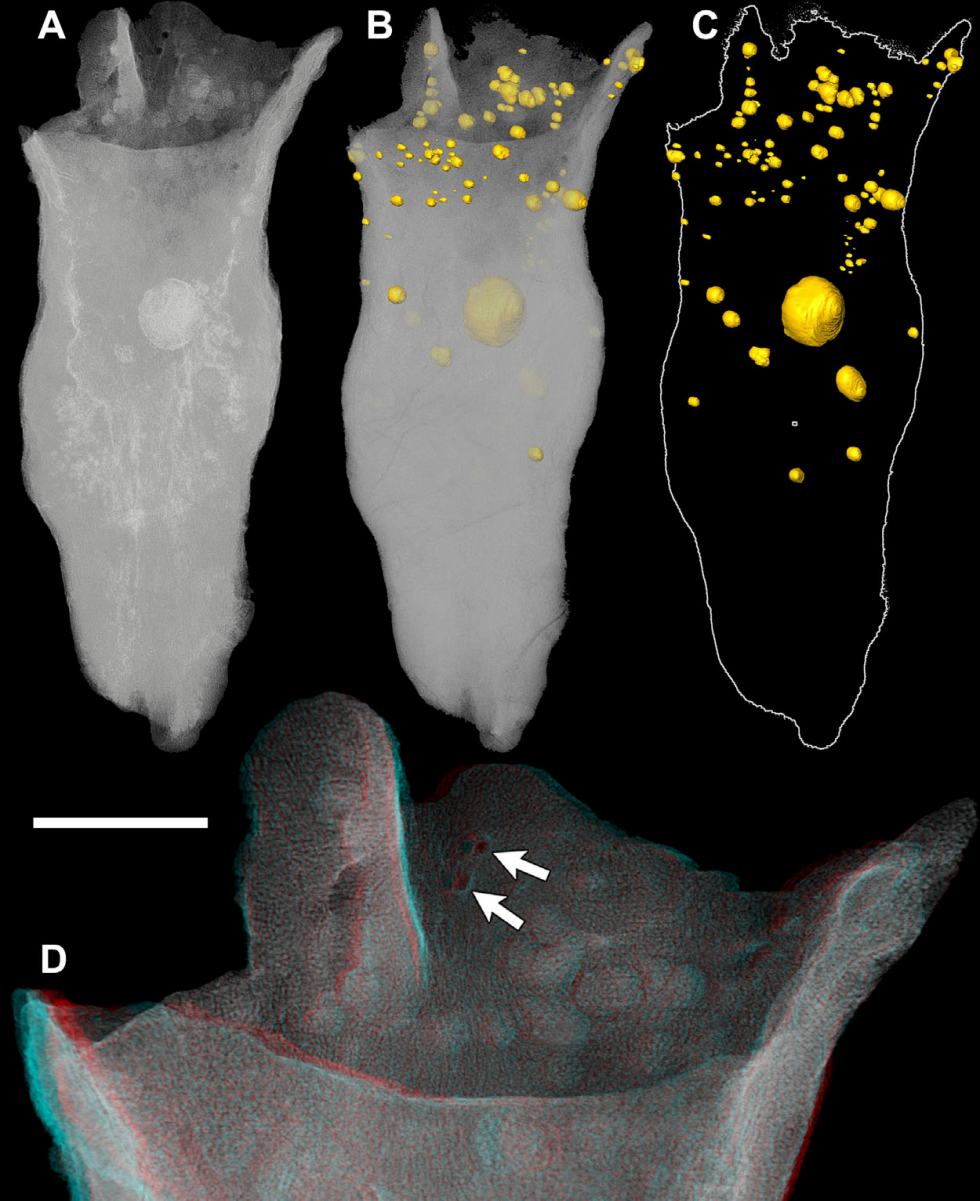
Reconstructions of the new conical organism (X 5331) based on SRXTM data. (A) Volume rendering. (B) Volume rendering with cell clusters reconstructed in yellow. (C) Reconstruction of the cell clusters. (D) Volume rendering of the aperture in stereo (red/blue anaglyph); white arrows indicate hemispherical hollows. Scale bar: (A–C) 300 μm, (D) 100 μm.

The single fossil has an irregular hollow conical form. It is 1.3 mm in length and diverges from the apex to an aperture having a diameter of 0.5 mm (Fig. 1A–D). It does not expand regularly from apex to aperture, but bulges at about one third of the length from the apex before narrowing and then flaring out to its widest point at the aperture. The apex is open with an angular and irregular margin, whereas the aperture has an irregular scalloped margin. The surface is covered with a series of hemispherical bulges and depressions ranging from 10 to 120 μm in diameter (Fig. 1A and B; Fig. 2). These occur on both the exterior and the interior (Fig. 1C, Fig. 3) of the hollow cone, though they are most abundant on the exterior margin close to the aperture. The largest bulge is on the interior.

Polygonal structures are visible on the exterior surface of the specimen (Fig. 2A–C). The SRXTM data reveal that these polygonal structures reflect the boundaries between cells that tend to lack preserved walls at the surface and make up the interior of the specimen (Fig. 1E–H). They are up to ∼5 μm in maximum dimension and have a mean volume of 17 μm^3^ (based on volumetric measurements of 20 cells). The cells are preserved with a low‐attenuation mineral phase at the cell boundaries. In most parts of the specimen the cells appear to be preferentially aligned, though the orientation varies through the specimen. In some regions there is a strong alignment of elongated cells into rows (Fig. 1E), whereas in most regions the organization is much weaker. The entire cone is made up of cells that are packed closely together. There are no canals passing through the structure.

The hemispherical bulges visible on the surface of the specimen can be seen to correspond to spherical clusters composed of up to thousands of cells (Fig. 1F–H; Fig. 3A–D). The mean size of cells in these clusters is larger than those in the rest of the body (mean of 60 cells from three clusters is 30 μm^3^). The cells in the largest cluster (cluster volume is 583,826 μm^3^; mean of 20 cells is 26 μm^3^) are slightly smaller than those in two smaller clusters measured (cluster volume 4836 μm^3^, mean of 20 cells 38 μm^3^; cluster volume 26,382 μm^3^, mean of 20 cells 40 μm^3^). Most of the cell clusters protrude from the margin of the specimen at least to some degree but some are completely surrounded by other cells. The clusters can be distinguished from the rest of the cellular mass by having higher X‐ray attenuation in their cell interiors than the cells that surround them (Fig. 1F–H). The regions adjacent to the clusters are composed of small, flattened cells that are orientated tangentially to the margin of the clusters (Fig. 1G). Where the clusters protrude from the surface, a layer of flattened cells lies between the clusters and the body cells but this does not extend over the protruding part of the cluster (Fig. 1H).

The hollows visible from the external surface range from 10 to 90 μm in diameter. A few craters (∼5) are visible on the internal walls, but they are much less common than on the outer walls. The ESEM data show that the concave surfaces of some of the craters preserve cells that are open (Fig. 2B and C), whereas others have relatively smooth surfaces (Fig. 2B and D). One of the relatively smooth hollows preserves a polygonal network of ridges (Fig. 2D).

## INTERPRETATION

We interpret the conical morphology of the organism as an original feature. The cone is composed of tessellating cells throughout, providing evidence that the cells are in life position and against an interpretation as an originally flat structure that has been rolled up. It is also unlikely that the conical morphology is an artifact of decay, mineralization or subsequent sedimentary abrasion. The approximately constant thickness around the diameter of the cone suggests that the conical morphology is an original feature. This is supported by the preservation of undistorted surface structures including the convex cell clusters and the concave craters. We interpret the irregular margin at the base as being due to breakage. This implies that the cone was attached to a substrate, either standing erect on the seafloor or protruding from a larger object, which may have been part of the same organism. In any case, the conical form entails a large surface area, which would promote exchange of nutrients with the surroundings or release of numerous propagules.

Within the cell clusters, the cell size is in the same order of magnitude for a cluster with approximately 130 cells and one with approximately 23,000 cells. If the differently‐sized cell clusters reflect successive stages in a developmental series, then this observation would suggest that the clusters did not undergo palintomy, but rather that cells increased in volume between divisions. As the craters are closely comparable in terms of size and shape to the cell clusters, they most likely represent scars formed when clusters left the organism.

The fact that smooth surfaces seen in some craters (Fig. 3D) are not seen in the rest of the specimen suggests that these preserve evidence of biological structures. It is possible that they represent envelopes that surrounded the cell clusters as they erupted. The polygons present on some of these surfaces do not appear to correspond to the cells of an erupted cluster as they can be over 10 μm in width and are therefore considerably larger than the cells. The ridges could represent original features of an envelope that surrounded a cluster, or they could be formed by deformation.

The discrete cell clusters might be interpreted as part of the conical organism. The preferential alignment of the cells around the clusters might provide some support for this interpretation, but could be the result of mechanical response to the expanding cluster volume. Alternatively, the clusters might be interpreted as tumors within the organism, or as exogenous saprophytic, parasitic, mutualistic or commensal organisms. A tumor interpretation seems unlikely for a number of reasons. Firstly, cancerous cells are generally irregular in size and shape, and tend to occur in clusters that have poorly defined boundaries in contrast to what is observed in this organism. Secondly, the large number of small clusters might be interpretable as secondary metastatic tumors, but metastasis is extremely rare outside vertebrates (Doonan and Sablowski 2010; Robert 2010). Thirdly, the evidence that the cellular bodies leave the organism seems at odds with this interpretation, as tumors are not normally transmitted between individuals. The cell clusters could be interpreted as saprophytes or parasites that infested the conical organism or as organisms that had mutualistic or commensal relationships with the conical organism. A saprophytic interpretation is unlikely as there is no evidence that the clusters consumed the cells around them or that these cells had decayed to a greater extent than the clusters themselves. However, we are unable to determine whether these structures represent parasitic, mutualistic or commensal organisms, or whether they form part of the conical organism.

**Figure 4:**
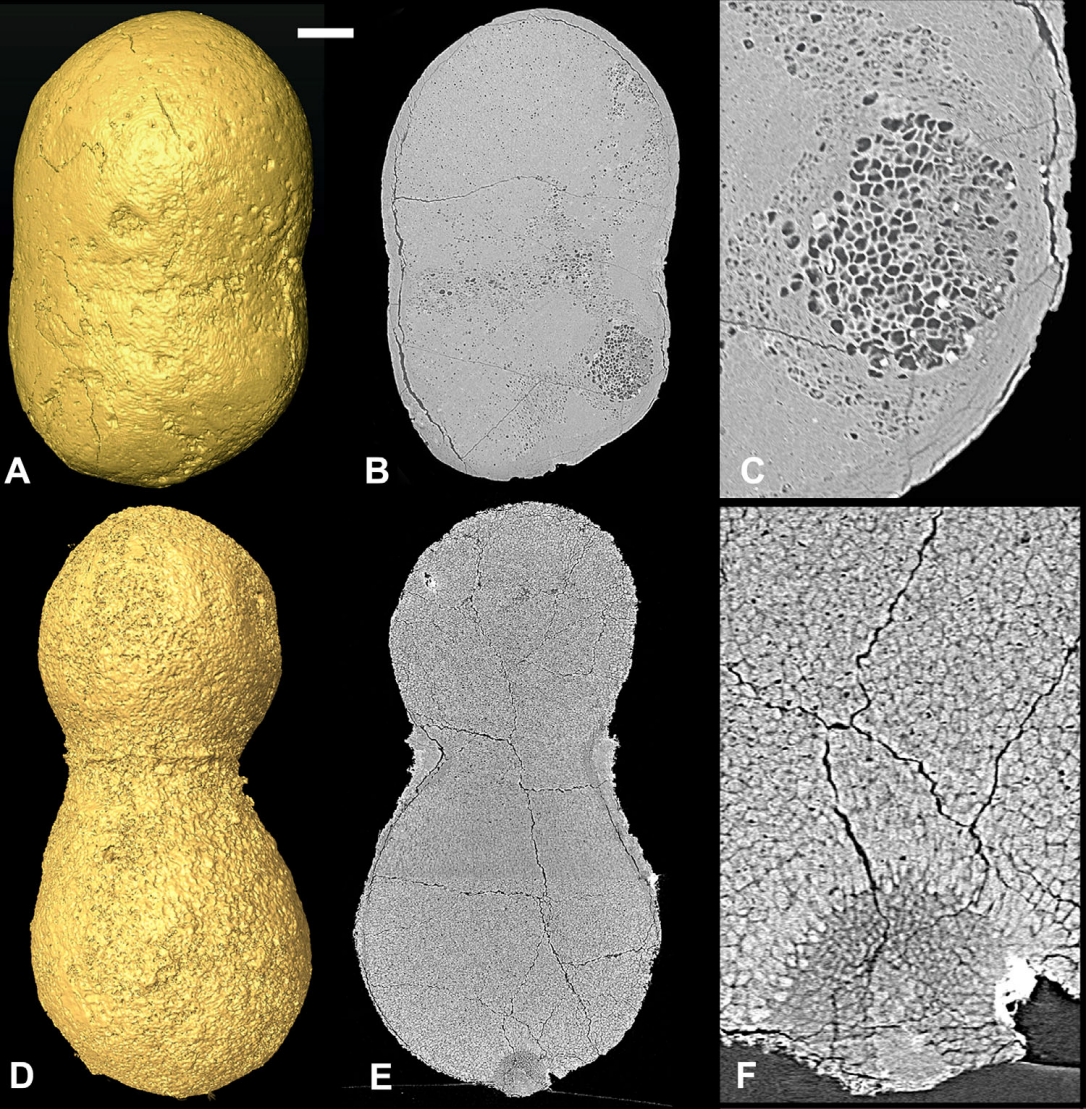
Reconstructions of peanut‐shaped fossils containing cell clusters based on SRXTM data. (A–C) X 5357. (A) Surface rendering. (B) Longitudinal slice. (C) Detail of “B” showing a cell cluster. (D–F) X 4447 (previously figured by Huldtgren et al. (2011). (D) Surface rendering. (E) Longitudinal slice. (F) Detail of “E” showing a cell cluster. Scale bar: (A,B) 125 μm. (C) 36 µm, (D,E) 100 μm, and (F) 20 μm.

The cell clusters could be interpreted as propagule‐like reproductive structures. This interpretation implies a life cycle where spherical clusters with a wide range of sizes and up to thousands of cells were released from the entire surface of the conical organism, including from the inside of the cone and not from specialized reproductive organs. As the propagule‐like structures were ejected, they left craters at their former position. We know of no living organism with a closely comparable reproductive mode. The cellular structures do, however, bear a close resemblance to other structures reported from the Doushantuo biota. These include structures preserved in purported red algae (Zhang and Yuan 1992; Xiao et al. 2004; Xiao et al. 2014), structures observed in the “Megaclonophycus”‐stages of *Tianzhushania*, which have also been reported as reproductive structures (Chen et al. 2014), and small cell clusters described in peanut‐shaped fossils with hundreds of thousands of cells (Huldtgren et al. 2011). Though these clustered cells in the peanut‐shaped fossils are not embedded in a thallus, they may occur in a thallus‐like mass of poorly defined cells. There is also similarity to larger structures observed in the peanut‐shaped‐forms (Fig. 4). This might suggest a common developmental strategy, but it might also be the result of infestation by similar exogenous organisms.

The number of cells in the organism and their organization into a conical form demonstrates a level of complexity that exceeds that found in any prokaryote. A wide variety of eukaryotes, including many animals (Hughes 1989), reproduce through cellular propagules. However, within animals, a eumetazoan interpretation for this fossil can be discounted, as the body composed of many thousands of cells, yet lacking distinct tissues, is incompatible with eumetazoans. The hollow conical form of the organism is superficially reminiscent of an archetypal sponge body plan. However, the organism is composed of a solid volume of cells that are tightly packed together. There are no intervening canals, which would be visible were they present given the fidelity of preservation and the resolution of the imaging technology. This means that the organism could not have functioned as a sponge. There is also little similarity to other early‐branching animal clades such as ctenophores and placozoans. We can therefore exclude a crown‐group animal interpretation for this specimen. The presence of Y‐shaped junctions between cells has been used to argue for an animal affinity for *Tianzhushania* (Xiao et al. 2012). Such junctions are also present in this specimen, but as they also occur in other groups this is insufficient to infer an animal affinity (Huldtgren et al. 2011, 2012). Although we cannot definitively reject the possibility that it is a stem‐group animal, there is no evidence to support this placement.

The fossil is safely interpreted as a eukaryote. However, it lacks characters to enable its affinities to be resolved precisely to any particular group. A placement within the non‐metazoan holozoans (which have also been compared to *Tianzhushania* (Huldtgren et al. 2011)) is possible, but there are no apomorphic characters to link the fossil to this group with confidence. Various clades of algae might be potential candidates, particularly the rhodophytes, phaeophytes, and chlorophytes, all of which have evolved thalli with large numbers of cells (Xiao et al. 2004). Many algae release multicellular vegetative propagules of varying morphology, some of which are similar to the cell clusters observed in the fossil (Cecere et al. 2011). Rhodophytes (red algae), which have already been reported from the Doushantuo assemblage (Xiao et al. 2004), provide the closest comparisons. For example, the rhodophyte *Gracilaria* has been observed to release spherical propagules from multicellular disks that originated as carpospores (Polifrone et al. 2006). Even more similar is the crustose freshwater red alga *Hildenbrandia* *angolensis*, which releases cylindrical gemmae that form internally and are ejected from the surface leaving craters resembling those seen in the conical fossil (Sherwood and Sheath 2000). There are differences however. For example, the propagules in *H. angolensis* are more uniform in size (48.6–71.0 μm in diameter) and are composed of branched or unbranched filaments. Furthermore, the overall morphology of this crustose alga is very different to that of the conical fossil. Nonetheless, the similarities between the fossil and these algae indicate that an interpretation as a reproductive thallus is plausible, regardless of the organism's precise phylogenetic affinity.

## COMPARISON WITH OTHER DOUSHANTUO TAXA

**Figure 5:**
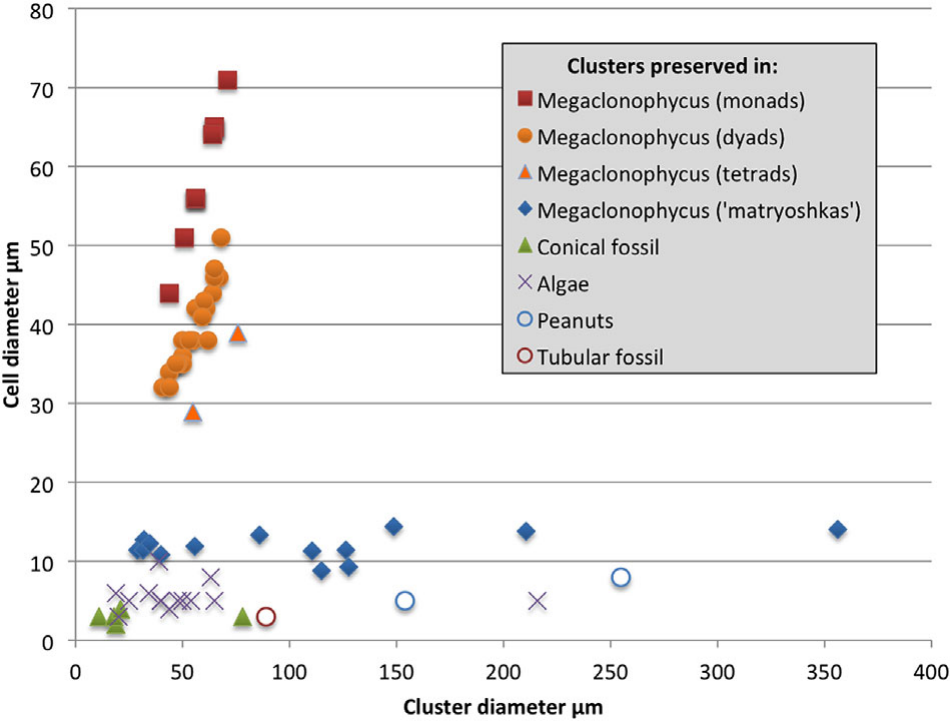
Plot of cell diameter against cluster diameter for cell clusters in Doushantuo fossils. Each data point represents a single cluster. Data for “matryoshkas” were measured by Chen et al. (2014); the remaining measurements are new. Cluster diameter was calculated as the mean of the maximum and minimum dimensions from 2‐D sections. Cell diameter was estimated from the mean of the maximum and minimum dimensions in 2‐D sections averaged over all visible cells for monads, dyads and tetrads and over five cells for larger clusters. *Megaclonophycus* monads, dyads and tetrads = one, two and four‐celled clusters respectively from *Megaclonophycus*‐stage specimens figured by Chen et al. (2014); *Megaclonophycus* “matryoshkas” = clusters from *Megaclonophycus*‐stage specimens measured by Chen et al. (2014); Conical fossil = clusters from the conical fossil described here; Algae = clusters in fossils interpreted as algae in Xiao et al. (1998), Xiao et al. (2004) and Xiao et al. (2014); Peanuts = clusters in new peanut‐shaped fossils; Tubular fossil = cluster in the tubular fossil in Figure 3d of Cunningham et al. (2015). Measurements are given in the Table S1.

Measurements of cell clusters found in the conical fossil and clusters in various other Doushantuo taxa are presented in Figure 5. These show that the clusters in *Megaclonophycus*‐stage specimens comprised of hundreds of cells, putative red algae and the conical fossil all maintain a constant cell size during growth. This indicates a similar mode of growth in each of these types of fossil. However, the absolute size of cells varies between each type of fossil, with the clustered cells in the conical fossil being distinctly smaller than the clustered cells in *Megaclonophycus*‐stage specimens. Clusters in *Megaclonophycus*‐stage specimens have the largest cells (mean = 11.9 μm), followed by those in algae (mean = 5.5 μm) and, finally, those in the new conical form (mean = 3.0 μm). The monads, dyads, and tetrads in *Megaclonophycus*‐stage specimens follow a pattern that is indicative of palintomic division. This is distinct from the pattern seen in the remaining clusters including those clusters in *Megaclonophycus*‐stage specimens that are interpreted as later developmental stages of the monads, dyads and tetrads (Chen et al. 2014).

The findings have implications for understanding the embryo‐like fossils from the Weng'an biota. The cell clusters in the conical fossil resemble cell clusters that have been reported from *Megaclonophycus*‐stage embryo‐like fossils with thousands of cells and have been presented as evidence for apoptosis and for differentiation between the germline and soma (Chen et al. 2014). Moreover, the interpreted presence of these characters has been used to support the possibility that these fossils are stem‐animals that had gained some, but not all, of the characters present in animals but not choanoflagellates (Chen et al. 2014). This is unfortunate as the interpretations of cell sorting, apoptosis, and cell differentiation, are entirely inferential and open to alternate interpretations of the evidence. Further, Chen et al. (2014) did not fully explore the possibility that the cell clusters are exogenous in origin—an hypothesis that we similarly cannot reject for the cell clusters in the conical fossil. This possibility should be revisited for the embryo‐like fossils. The sole line of evidence against an exogenous origin for these structures in the embryo‐like fossils is a possible continuum between the clusters and blastomere‐like monads and associated dyads and tetrads. However, the monads, dyads and tetrads underwent palintomy—in contrast to the pattern observed in the cell clusters—reducing support for the hypothesis of a developmental continuum between the monads, dyads, and tetrads on the one hand, and the cell clusters on the other (Tang 2015). With it, support for an endogenous origin of the clusters is weakened concomitantly. Alternatively, indefinite palintomy is unsustainable and a switch from this mode of division is required at some stage (Tang 2015; Chen et al. 2015). Some support for this scenario comes from the measurements presented in Figure 5, where the L‐shaped pattern expected under a switch from palintomy at some point between the 16‐cell‐stage and the 256‐cell‐stage (depending on cluster diameter) is observed. Nevertheless, there is a lack of intermediates between the tetrads and “matryoshkas” and it is necessary to establish a stronger test of these competing hypotheses because the key interpretations of the fossils (Chen et al. 2014) rely on an endogenous nature of the cell clusters described as “matryoshkas.” The alternative possibility, that the “matryoshka” cell clusters in the embryo‐like fossils and the cell clusters in the conical fossil represent infestation by exogenous organisms, would mean that the structures have no bearing on our understanding of the affinities or life cycle of the host. This hypothesis must therefore be rejected before the “matryoshka” cell clusters can be marshaled as evidence of endogenous cell differentiation, supporting an animal interpretation. Alternative possibilities are that the shared presence of these cell clusters evidences a developmental or phylogenetic link between the conical fossil and the embryo‐like fossils. The difference in size between the cell clusters in the conical fossil and the “matryoshkas” in the embryo‐like fossils suggests that a direct ontogenetic connection is unlikely. Nevertheless, either of these alternative interpretations would also speak against an interpretation as animals.

## CONCLUSIONS

We present a new Doushantuo organism with a conical body that is preserved at a cellular level. The body contains cell clusters as well as craters that are interpreted as scars formed where the clusters left the organism. Similar cell clusters are found within a variety of Doushantuo taxa including embryo‐like fossils, peanut‐shaped forms, algae and the conical organism described here. These features may represent a developmental stage in the lifecycle of the organisms. If so, this implies a similar lifecycle in the various taxa. On the other hand, the cell clusters might be exogenous organisms that are parasitic, mutualistic or commensal. If this latter interpretation is correct, this suggests that different Doushantuo taxa host similar exotic organisms. Gaining a better understanding of the Doushantuo fossils and their affinities will require study of the diverse irregular forms within the deposit and the life cycles of the Doushantuo organisms.

## Supporting information

Additional supporting information may be found in the online version of this article at the publisher's web‐site.

**Table S1**. Tubular fossil Cunningham et al. (2015) Figure 5.
